# Genome analysis of *Pseudomonas syringae* pv. *actinidiae* biovar 6, which produces the phytotoxins, phaseolotoxin and coronatine

**DOI:** 10.1038/s41598-019-40754-9

**Published:** 2019-03-07

**Authors:** Takashi Fujikawa, Hiroyuki Sawada

**Affiliations:** 1 Institute of Fruit Tree and Tea Science, National Agriculture and Food Research Organization (NARO), Fujimoto 2-1, Tsukuba, Ibaraki 305-8605 Japan; 2 Genetic Resources Center, NARO, Kannondai 2-1-2, Tsukuba, Ibaraki 305-8602 Japan

## Abstract

The kiwifruit bacterial canker pathogen, *Pseudomonas syringae* pv. *actinidiae* (Psa), causes enormous economic damages in many kiwifruit producing countries. In 2015, biovar 6, the novel biovar of Psa, was found in Nagano Prefecture, Japan. The genomes of two representative strains of biovar 6 (MAFF 212134 and MAFF 212141) were sequenced and analysed, indicating that their genomes are the most similar to that of biovar 3 among the known Psa biovars, based on average nucleotide identity analysis. Biovar 3 has neither the phaseolotoxin synthesis gene cluster nor the coronatine synthesis gene cluster, whereas biovar 6 has both clusters and produces both phytotoxins. We found that biovar 6 possesses 29 type III secreted effector (T3SE) genes, among which *avrRps4* and *hopBI1* are unique to biovar 6. The expression of T3SE genes and two phytotoxin synthesis gene clusters of biovar 6 during the early stages of host infection was investigated using RNA-Seq analysis, showing that these genes could be grouped into three categories: constantly expressed genes, constantly suppressed genes, and temporarily induced genes. A PCR assay was established to differentiate biovar 6 strains from the other Psa biovars and the closely related pathovar, pv. *actinidifoliorum*, by using *avrRps4* as a biovar 6-specific marker gene.

## Introduction

Kiwifruit, a type of deciduous fruit tree, belongs to the genus *Actinidia* and is cultivated commercially in many parts of the world, and its economic importance has been increasing in recent years. However, the spreading of kiwifruit bacterial canker disease has been widely observed recently, resulting in serious economic losses and, in some cases, reduced cultivation of kiwifruit^1,2^. The following are representative symptoms of the disease: necrotic spots on leaves, canker and wilting on canes and twigs, dieback of trunks, with a red or milky white exudate.

The causal agent of this disease is *Pseudomonas syringae* pv. *actinidiae* Takikawa, Serizawa, Ichikawa, Tsuyumu and Goto 1989 (Psa). Psa has been found in many of the major kiwifruit production areas of the world^1^. Based on genetic diversity and toxin production^3–5^, Psa had been categorized into six biovars (biovars 1–6). However, recently biovar 4 has been transferred to another pathovar, *actinidifoliorum* (Pfm)^6^, so that Psa is now composed of five biovars shown below. Biovar 1, including the Japanese isolates that were described as Psa for the first time in the world^7^, can produce phaseolotoxin, a type of phytotoxin^8^. In Japan, biovar 1 has been found to be distributed widely^7,9^. Biovar 2, which has been found only in South Korea so far, can produce coronatine, another type of phytotoxin. Biovar 3 is a severe virulent group, causing pandemics throughout the world. However, it has not been clarified so far that biovar 3 produces any known toxins^9^. Biovar 5 was recorded in Japan in 2014, and like biovar 3, the ability to produce known toxins has not been confirmed from this biovar^4,5,9^. Since biovar 5 is confirmed only in Saga Prefecture located on Kyushu Island, Japan^4^, it is considered to be an endemic lineage.

In 2015, biovar 6 was discovered in Japan, but its distribution was confirmed to be limited to a small part of Nagano Prefecture on Honshu Island^10^, suggesting that biovar 6 may also be an endemic lineage similar to biovar 5. However, in contrast to biovar 5, biovar 6 can produce two phytotoxins, phaseolotoxin and coronatine^10^. Although there have been numerous studies on phytotoxins to date, no phytopathogenic bacteria that produce more than one phytotoxins have been investigated. Therefore, studying the genetic characteristics of biovar 6 is important for understanding the evolutionary paths and pathogenic mechanisms of phytotoxin-producing bacteria. Here, we sequenced the genomes of biovar 6, and compared them with those of the other Psa biovars and Pfm, especially focusing on the loci responsible for the synthesis of phytotoxins and type III secreted effectors (T3SEs), which are considered as major factors involved in pathogenicity/virulence. Moreover, we investigated gene expression of biovar 6 upon its host infection and established a PCR assay to identify biovar 6 specifically.

## Results

### Biovar 6 genome information

The genomes of strains MAFF 212134 and MAFF 212141, selected as representative strains of biovar 6, were sequenced. Using an Ion PGM next generation sequencing system (Thermo Fisher Scientific Inc., Waltham, MA, USA), 5,435,332 single reads with average length of 273.6 bp (MAFF 212134), and 5,744,779 single reads with average length of 287.5 bp (MAFF 212141) were acquired, respectively. Genomic *de novo* assembly was achieved using a CLC Genomics Workbench (Qiagen, Valencia, CA, USA). After filtering to remain only high quality reads (reads of <20 at a Phred score were cut-off), 350 contigs in MAFF 212134 (>500 bp with an N_50_ of 38,456 bp) and 334 contigs in MAFF 212141 (>500 bp with an N_50_ of 37,734 bp) were acquired (GenBank accession numbers MSBW00000000 and MSBX00000000). As a result, the assembled genome size of MAFF 212134 was 6,021,851 bp (genome coverage is a 246.95-fold), while that of MAFF 212141 was 6,033,395 bp (genome coverage is a 273.75-fold). Both genomes had a guanine-cytosine (GC) content of 58.7%. The assembled contig sequences of MAFF 212134 were annotated using NCBI Prokaryotic Genome Annotation Pipeline (PGAP). Consequently, 5,796 protein-coding DNA sequences (CDSs), 52 tRNA genes and four rRNA genes were identified. Similarly, the assembled contig sequences of MAFF 212141 were also annotated using NCBI PGAP, and 5,841 CDSs, 50 tRNA genes and five rRNA genes were identified. The sequences of *acnB*, *cts*, *gapA*, *gyrB*, *pfk*, *pgi*, and *rpoD*, which are well-known as housekeeping genes and have been used as molecular markers for phylogenetic analyses, were extracted from the genome sequences of MAFF 212134 and MAFF 212141 determined in this study and compared with those of the respective strains reported in our previous study^10^, showing that they are completely identical.

### Average nucleotide identity

For primarily taxonomic purposes, genome similarity has been determined by performing whole genome DNA-DNA hybridisation experiments between bacteria. In recent years, however, instead of wet experiments, *in silico* (dry) analysis is more commonly used, because it is simple and accurate. The average nucleotide identity (ANI) analysis is extensively used as a representative of such *in silico* analysis^11^. Actually, in our previous study, the genome similarity of Psa including biovar 5 could be clarified by ANI analysis^5^.

**Figure 1:**
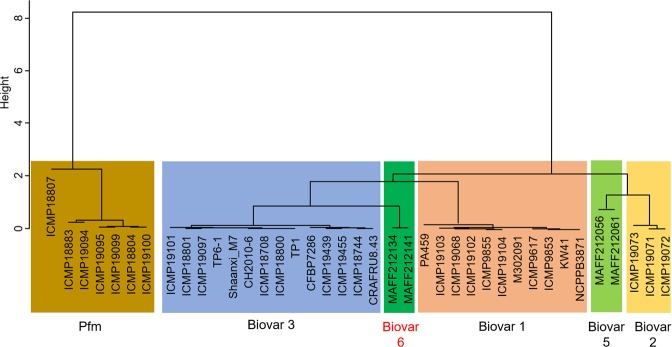
Dendrogram of genome similarity based on ANI values. A dendrogram based on ANI values was constructed using UPGMA. Lineage 1 (ICMP 18883, 18804, 19094, 19095, 19099, 19100) and lineage 3 (ICMP 18807) of *P. syringae* pv. *actinidifoliorum* (Pfm) were included in this analysis (lineages in Pfm are considered to be equivalent to biovars in Psa).

ANI values among Psa biovars including biovar 6 and Pfm (Suppl. Table 1), were acquired using the ANI calculator; the results showed that the reciprocal values between the two strains (MAFF 212134 and MAFF 212141) of biovar 6 used in this study completely matched (100% identity). These two strains of biovar 6 had 99.59–99.63% identity with biovar 1, 99.41–99.43% identity with biovar 2, 99.82–99.85% identity with biovar 3, 99.39–99.40% identity with biovar 5, and 98.17–98.25% identity with Pfm. Thereafter, a dendrogram based on ANI values was constructed by using the unweighted pair group method with arithmetic mean (UPGMA); the results showed that the two strains of biovar 6 clustered independently of the other Psa biovars and Pfm, and that biovar 3 was the sister group to biovar 6 (Fig. 1). Pfm strains were grouped together and were positioned apart from Psa biovars entirely. When other algorithms (e.g. nearest-neighbour chain algorithm method, ward method, and complete-linkage clustering method) were used, similar topologies of dendrograms were obtained (data not shown). These topologies also agreed well with that of the phylogenetic tree constructed based on multilocus sequence analysis (MLSA) in our previous study^10^.

### Phytotoxin biosynthesis gene clusters

The phytotoxins, verified so far to be produced by Psa, are phaseolotoxin and coronatine^1,2,8^. It has been experimentally confirmed that biovar 1 and biovar 2 produce phaseolotoxin and coronatine, respectively, while none of known phytotoxins have been detected from biovars 3, 5 and Pfm so far^1,2,4–6,9^. Although gene cluster-like structures that could be involved in the production of unidentified non-ribosomal peptides or uncharacterized secondary metabolites were found in Psa and Pfm genomes (data not shown), it has not been experimentally proved yet whether these products actually have the function as a phytotoxin. On the other hand, biovar 6 certainly produces both phaseolotoxin and coronatine^10^. By determining the draft genomes of biovar 6, we clarified genes involved in the biosynthesis of these phytotoxins.

**Figure 2:**
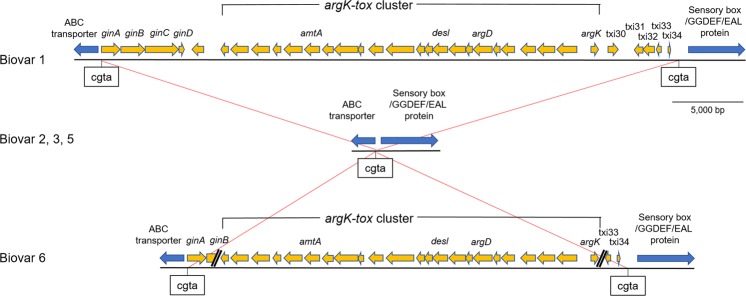
Comparative analysis of the *tox* island region in each biovar. Results of the comparative genome analysis are schematically presented. Locus of the *tox* island, a genomic island which contains *argK-tox* cluster (phaseolotoxin synthesis gene cluster)^12^, and its flanking regions in biovar 1 (upper) and biovar 6 (lower) are shown. The corresponding loci in biovars 2, 3, and 5 (middle) are also presented. Box arrows indicate predicted genes and their directions. Orange arrows correspond to the *tox* island and blue arrows correspond to its flanking regions.

The *tox* island containing the *argK-tox* cluster (phaseolotoxin synthesis gene cluster) is located on the chromosomes of phaseolotoxin-producing pathogens, Psa biovar 1 and *P. syringae* pv. *phaseolicola*^12^. Several enzyme-coding genes, such as *amtA*, *desl*, *argD* and *argK*, of the *argK-tox* cluster (Fig. 2) were already confirmed to be present in biovar 6 as well by PCR assays^10^. In this study, by using comparative genome analysis, we found that the *tox* islands in biovar 1 and biovar 6 are inserted in the same direction at the homologous positions of their chromosomes (Fig. 2); namely, the *tox* island of biovar 6 is inserted in the ‘CGTA’ sequence site^12^ between the coding regions of the ABC transporter and sensory box/GGDEF/EAL protein, in the same way as biovar 1. However, in both end regions of the *tox* island, a slight difference was recognised between these biovars; several genes are lost in the regions of biovar 6 (Fig. 2). Especially, *ginABCD* operon, which is located at the left end of the *tox* island and is involved in excision/insertion of the *tox* island^13^, was found to be truncated, suggesting that the island of biovar 6 might have lost its mobility.

**Figure 3:**
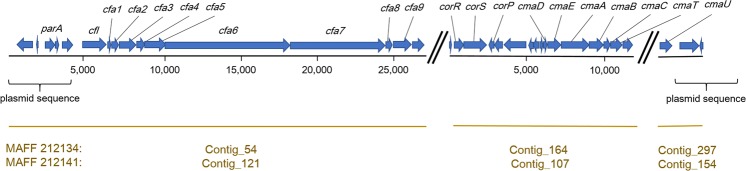
Coronatine synthesis gene cluster of biovar 6. Results of the comparative genome analysis are presented. Locus of the coronatine synthesis gene cluster and its flanking regions, consisting of three contigs of biovar 6, are shown. Box arrows indicate predicted genes and their directions.

In coronatine-producing pathogens such as Psa biovar 2, *P. syringae* pv. *glycinea*, and *P. syringae* pv. *tomato*^14,15^, the coronatine synthesis gene cluster, consisting of coronafacic acid (CFA) synthase genes (e.g. *cfl* and *cfa1*) and coronamic acid (CMA) synthase genes (e.g. *cmaD* and *cmaU*), is conserved and is usually located on a megaplasmid. However, in some cases, the gene cluster has been confirmed as inserted on a chromosome. It was previously found that biovar 6 also possesses some of these genes by PCR assays, and certainly produces coronatine^10^. In the present study, we confirmed that the entire coronatine synthesis gene cluster is conserved in biovar 6, and that the flanking regions of the gene cluster correspond to plasmid-derived sequences, including the partition protein gene *parA* (Fig. 3), suggesting that it may be located on a plasmid just like biovar 2^16,17^.

### Type III secreted effector genes

It has been confirmed that type III secreted effectors (T3SEs), translocated to host plant cells via the type III secretion system (T3SS), play an important role in virulence/avirulence^18^. However, the composition of T3SE genes of biovar 6 has not yet been determined. In the present study, T3SE genes of biovar 6 could be listed by comparisons of the Psa genomes using the Mauve tool and/or translated BLAST (tBLASTx) analysis. However, unlike our previous study^5^, some effector genes—which are predicted to be *hrpL-*independent, or to have poor N-terminal signals for T3SS or no *hrp* box promoters upstream of the CDSs—were excluded from this list (Table 1). Finally, 29 T3SE genes were found in both genomes of strains MAFF 212134 and MAFF 212141 (Suppl. Table 2). The following genes corresponded to Psa/Pfm-conserved T3SE genes: *avrE*, *hopAA1-1*, *hopN1*, *hopS2*, *hopAS1*, *hopR1*, *hopAH1*, and *hopAZ1*, and the following genes corresponded to Psa-conserved T3SE genes: *avrD1*, *hopD1*, *hopQ1*, *hopY1*, *hopZ3*, *avrPto5*, and *hopAU1*. Although *hopI1* was conserved in Psa biovars 1, 2, 3, 5, and Pfm, only biovar 6 was found to lack it. On the other hand, *avrRps4* and *hopBI1* were found to be unique to biovar 6. In MAFF 212134, *avrRps4* (BUE60_15715) was located on Contig_91 of its genome, and in MAFF 212141, *avrRps4* (BUE61_17920) was located on Contig_126 of its genome. Since both contigs were large in size (>40 kb), we were able to confirm that they possess some DNA maintenance genes, such as the partition protein gene *parA* and the plasmid stability protein gene *stbB*, in addition to *avrRps4*, suggesting that each contig carrying *avrRps4* may be derived from a plasmid possessed by biovar 6. Regarding *hopBI1*, it was located on Contig_339 of MAFF 212134 (BUE60_29210), and on Contig_307 of MAFF 212141 (BUE61_29115). Because these contigs were short in length, it was unclear whether *hopBI1* is located on the chromosome or on a plasmid.

**Table 1:** Predicted type III secreted effector genes of biovar 6; biovars 1, 2, 3, and 5; and Pfm.

Genes^a^	Biovar 6 (MAFF 212134, MAFF 212141)	Biovar 1^b^	Biovar 2^b^	Biovar 3^b^	Biovar 5^b^	Pfm^b,c^ (Biovar 4)
*avrRpm1*	+	+	−	+	incomplete	−
*avrE*	+	+	+	+	+	+
*hopM1*	+	+	incomplete	+	+	+
*hopAA1-1*	+	+	+	+	+	+
*hopN1*	+	+	+	+	+	+
*hopI1*	−	+	+	+	+	+
*hopS2*	+	+	+	+	+	+
*hopBB1-1*	−	variable	incomplete	+	−	−
*hopAO2*	−	variable	−	+	−	−
*hopAF1-1*	−	variable	variable	+	+	+
*hopBB1-2*	+	variable	−	+	incomplete	−
*hopAW1*	+	incomplete	−	+	+	−
*hopX3*	−	variable	+	+	−	−
*hopAY1*	+	variable	+	incomplete	+	+
*avrB4*	+	variable	+	+	+	−
*avrD1*	+	+	+	+	+	−
*hopD1*	+	+	+	+	+	−
*hopQ1*	+	+	+	+	+	−
*hopF2*	−	variable	+	+	+	−
*hopAR1*	−	variable	−	−	−	+
*hopF1*	−	−	−	−	−	+
*hopAF1-2*	−	−	−	−	incomplete	+
*hopA1*	+	−	incomplete	incomplete	+	+
*hopY1*	+	+	+	+	+	incomplete
*avrRpm2*	−	incomplete	+	incomplete	+	−
*hopZ3*	+	+	+	+	+	−
*hopAS1*	+	+	+	+	+	+
*hopZ5*	−	−	−	+	−	−
*hopH1*	−	−	−	+	−	−
*hopAM1-1*	−	incomplete	−	+	−	−
*hopAE1*	+	+	variable	+	+	+
*hopW1*	+	incomplete	incomplete	incomplete	+	+
*hopR1*	+	+	+	+	+	+
*hopAG1*	−	incomplete	+	+	+	incomplete
*hopAH1*	+	+	+	+	+	+
*hopAI1*	+	incomplete	+	+	+	+
*hopAM1-2*	−	−	−	+	+	−
*avrPto5*	+	+	+	+	+	−
*hopAZ1*	+	+	+	+	+	+
*hopAV1*	+	variable	−	incomplete	incomplete	−
*hopAA1-2*	−	−	−	incomplete	−	−
*hopAU1*	+	+	+	+	+	−
*hopX1*	−	variable	−	−	−	+
*hopX2*	−	variable	−	−	−	+
*hopBD2*	−	+	incomplete	−	−	−
*hopH3*	−	+	−	−	−	−
*hopO1*	−	−	−	−	−	+
*hopT1*	−	−	−	−	−	+
*hopS1*	−	−	−	−	−	+
*hopE1*	+	−	−	−	−	+
*hopAB3*	−	−	−	−	−	+
*avrRps4* ^d^	+	−	−	−	−	−
*hopBI1* ^d^	+	−	−	−	−	−

^a^Type III secreted effector (T3SE) genes are predicted using the NCBI Prokaryotic Genome Annotation Pipeline (PGAP), the Mauve tool, and tBLASTx analysis in conjunction with the NCBI GenBank and Hop effectors public database (http://pseudomonas-syringae.org). Suspicious candidates as T3SE genes (*hrpL*-independent, poor T3SS N-term signal, or no *hrp* box) are excluded.

^b^Partial hits or truncated/disrupted sequences are indicated as ‘incomplete’. Also, referring to McCann *et al*.^21^, when the presence/absence of a gene is dependent on strains of the same biovar, it is indicated as ‘variable’.

^c^Psa ‘biovar 4’ had been transferred to the new pathovar *actinidifoliorum* (Pfm)^6^.

^d^The two T3SE genes at the bottom of the table, *avrRps4* and *hopBI1*, were found to be biovar 6-specific in this study.

As for the contigs carrying *avrRps4* (Contig_91 of MAFF 212134, Contig_126 of MAFF 212141), the sequences of the regions other than *avrRps4* were found to be similar to those of a plasmid possessed by *P. syringae* pv. *phaseolicola* 1448 A (E value = 0.0, identity = 89% according to nucleotide BLAST). On the other hand, as for the contigs carrying the coronatine synthesis gene cluster (Contigs_54, 164, and 297 of MAFF 212134, Contigs_121,107, and 154 of MAFF 212141), the sequences of the regions other than the cluster were similar to those of other bacterial plasmids such as *P. syringae* pv. *glycinea* plasmid p4180A and *P. syringae* isolate CFBP 3840 plasmid PP3 (e.g. E value = 0.0, identity = 99%). Considering these results, the plasmid carrying *avrRps4* seems to be different from the plasmid carrying the coronatine synthesis gene cluster.

### Expression of pathogenicity-related genes during the early stages of host infection

Various virulence factors of biovar 6, including T3SEs, phaseolotoxin, and coronatine, are assumed to be produced during host infection. Notably, genes expressed during the early stages of infection seem to be important, because these are presumably involved in bacterial invasion into host tissues, their establishment in the apoplasts and vascular spaces, suppression of host immunity, and invocation of pathogenicity^19^. When *A. deliciosa* ‘Hayward’ was spray inoculated with biovar 6 (MAFF 212134), typical lesions on leaves were observed to form gradually from one week after the inoculation (Suppl. Fig. 1). Here, gene expression of biovar 6 (MAFF 212134) during the early stages after inoculation was investigated using RNA-Seq analysis. Total RNAs extracted from leaves at 0, 4, 8, and 24 h after inoculation were examined. Each examination was carried out in six replicates. The raw data were submitted to the Sequence Read Archive (SRA) at NCBI under the BioProject PRJNA357960 (SRR8569152-SRR8569163 and SRR8569199-SRR8569210). Although these RNAs consisted of a large amount of plant RNAs in addition to a small amount of RNAs derived from biovar 6, RNA-Seq for biovar 6 could be performed using the biovar 6 genome sequenced in this study as the reference. Some sequence reads (ca. 1.2 × 10^4^ to 1.8 × 10^5^) derived from RNAs in each sample were mapped to the reference genome (ca. 5–40% of each sample RNA), and the mapped reads were regarded as the fragments of expressed genes of biovar 6 (Suppl. Table 3). After the gene expression value was calculated from the ratio of the number of mapped reads to reads per kilobase million (RPKM), the log_2_ fold-change against the 0 h sample was indicated as a differential expression value. In Suppl. Table 4, expressed genes with a false discovery rate (FDR) < 0.1 and the effect size (eta-squared), are listed. Here, when the sample parameter is small or the number of iterations is small, the p-value tends to be large^20^. And when expression comparison is performed for each gene at 0, 4, 8, 24 h, the p-value also becomes large due to many variables. Then, it is considered that the second type of error in statistical analysis (Type II error) is likely to occur. The occurrence of Type II error means to produce false negative results. Therefore, in order to avoid Type II error as much as possible, expressed genes were filtered at 0.1 of permissive FDR value, and the effect size (eta-squared) was calculated and attached on the statistical data of each gene expression.

Compared to 0 h after inoculation, the expression of various genes changed significantly or effectively. In Suppl. Table 4, 65 transcriptional regulator genes were included, which showed various expression patterns. The proportion of transcriptional regulator genes induced at 4 and/or 8 h after inoculation was found to be relatively high (Suppl. Fig. 2). In the T3SE genes, although expression of nearly half of the genes was suppressed, the expressed genes were found to be classified into several groups based on their expression patterns (Suppl. Table 5): only *hopAU1* showed constant expression through the early stages of infection; *hopAH1* was expressed at 4 h after inoculation; *hopAW1*, *hopAE1*, *hopAS1*, *hopY1*, *avrE*, *hopR1*, *hopAY1* and *hopBB1* were expressed at 24 h after inoculation; and expression of *hopQ1*, *hopZ3*, *hopAA1*, *hopS2*, *hopBI1* and *hopE1* varied throughout the early stages of infection. Regarding genes for the synthesis of the two phytotoxins, the expression of relatively many genes involved in phaseolotoxin synthesis tended to be suppressed in the early stages of infection, except for some genes such as *amtA* and dCTP deaminase gene (Suppl. Table 6), whereas approximately 40% of the coronatine synthesis genes were highly expressed at 4 and/or 8 h after inoculation (Suppl. Table 7, Suppl. Fig. 2).

### Biovar 6-specific identification

**Figure 4:**
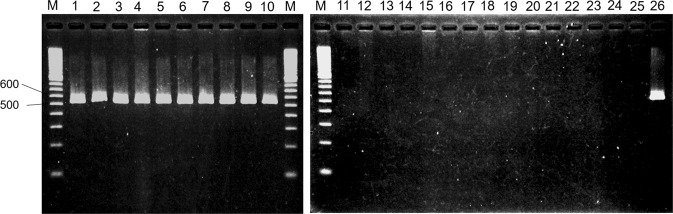
PCR analysis for *avrRps4* possession in Psa and Pfm. This figure consists of two electrophoresis gels (left gel shows two 100 bp DNA ladder marker lanes and lanes 1 to 10; right gel shows a 100 bp DNA ladder marker lane and lanes 11 to 26). M; 100 bp DNA Ladder, 1–10 and 26; biovar 6 (1, MAFF 212131; 2, MAFF 212132; 3, MAFF 212133; 4, MAFF 212134; 5, MAFF 212135; 6, MAFF 212136; 7, MAFF 212137; 8, MAFF 212138; 9, MAFF 212139; 10, MAFF 212140; 26, MAFF 212130), 11; biovar 1 (11, MAFF 211985), 12; biovar 2 (12, ICMP 19072), 13–17; biovar 3 (13, ICMP 18884; 14, ICMP 18744; 15, ICMP 19079; 16, ICMP 19439; 17, ICMP 19455), 18 and 19; biovar 5 (18, MAFF 212056; 19, MAFF 212057), 20 and 21; Pfm (Psa ‘biovar 4’) lineage 1 (20, CFBP 7812; 21, CFBP 8045), 22; Pfm lineage 2 (22, CFBP 8043), 23; Pfm lineage 3 (23, ICMP 18807), 24 and 25; Pfm lineage 4 (24, CFBP 7908; 25, CFBP 8041). The amplicons of *avrRps4* (530 bp) were obtained in only biovar 6 strains.

Because *avrRps4* is unique to biovar 6 among Psa biovars and Pfm (Table 1), it is expected that this gene may act as a biovar 6-specific marker. Thus, two PCR primers (*avrRps4*-F1 and *avrRps4*-R2) were designed on the basis of this sequence. It was confirmed that this primer set induced biovar 6-specific DNA amplification by means of a colony-direct PCR assay (Fig. 4). By electrophoresis, the amplicons derived from biovar 6 templates were found to be single bands (530 bp). In contrast, no amplification was detected when the other Psa biovars and Pfm were used as templates. Therefore, this primer set was found to be applicable to biovar 6-specific identification when identifying pathogens isolated from lesions of kiwifruit bacterial canker disease.

## Discussion

Biovar 6, the most recently described Psa biovar (at the time of 2019), was isolated in 2015 in a limited area of Nagano Prefecture^10^. Regarding the orchards where biovar 6 strains were isolated, it has been confirmed by interviews with local farmers that grafting scions, seedlings, and pollens imported from foreign countries have never been introduced in these orchards. Additionally, no occurrence of the other Psa biovars has been confirmed around the orchards. To the best of our knowledge, currently, there is no evidence to suggest the origin of this biovar. By the way, it is considered that *Actinidia* spp. are indigenous to Eastern Asia, and wild *Actinidia* plants are growing in fields and mountains in this area. And it is supposed that there are many bacteria, including *P. syringae*, colonising the phyllosphere, such as surfaces of leaves and flower buds, of *Actinidia* plants; thus, it is inferred that Psa might be derived from *P. syringae* strains coexisting with *Actinidia* spp. grown in these areas^21^. Therefore, in order to clarify the centre of origin and evolutionary paths of Psa, it is important to collect Psa strains derived from the diverse origins, analyse their genomes, and compare them with those of wild *P. syringae* strains.

In the present study, the genomes of MAFF 212134 and MAFF 212141 (as the representative strains of biovar 6) were sequenced, and genome similarity based on ANI values was investigated; the results clearly showed that the genome of biovar 6 is the most similar to that of biovar 3 among the existing Psa biovars (Fig. 1, Suppl. Table 1). The topology of the dendrogram based on ANI values (Fig. 1) agreed well with that of the phylogenetic tree which was constructed based on MLSA using seven housekeeping genes in our previous study^10^. These results suggest that the genomic structures are roughly similar between biovar 6 and biovar 3. However, in contrast to the overall structure of their genomes, the repertoires of several genes related to the pathogenicity/virulence, especially the phytotoxin synthesis gene clusters and the T3SE genes, were found to be fairly different between the two biovars in the present study.

Intriguingly, biovar 6 actually produces both phaseolotoxin and coronatine^10^. On the other hand, besides biosynthesis gene clusters for phaseolotoxin and coronatine, some gene cluster-like structures, that might be involved in the production of unidentified non-ribosomal peptides or uncharacterized secondary metabolites, were found in the genomes of Psa biovars, including biovar 6, and Pfm (data not shown). However, none of these products have been identified as phytotoxins as yet through experiments. Thus, to the best of our knowledge, except for Psa biovar 6, no phytopathogenic bacteria have ever been experimentally confirmed to produce more than one phytotoxins, showing that study of this biovar may aid in elucidating the evolutionary paths and pathogenic mechanisms of phytotoxin-producing bacteria.

In our previous study, a gene cluster with a size exceeding 25 kb was confirmed to be necessary for *P. syringae* (Psa biovar 1 and *P. syringae* pv. *phaseolicola*) to synthesise phaseolotoxin^12^. This gene cluster is called the *argK-tox* cluster (Fig. 2), and is contained in a genomic island (*tox* island)^12^. We previously indicated that this *tox* island was originally located on a chromosome of another bacterium other than *Pseudomonas*, from which it was horizontally transferred and integrated site-specifically into *P. syringae* chromosomes, based on the following grounds: the phylogenetic positions of some CDSs contained in the *tox* island are far from those of their respective homologues located on *P. syringae* chromosomes; the *tox* island also contains three CDSs (*ginA, B, C*), which encode tyrosine recombinases (site-specific recombinases), in addition to the *argK-tox* cluster; and the codon usage, GC content, and GC content at the codon third position of CDSs contained in the island are remarkably different from those of *P. syringae* chromosomes^8,12,22,23^. Furthermore, in the present study, comparative genome analysis of biovar 1 and biovar 6 showed that their *tox* islands are both inserted in the same direction at the homologous positions of the respective chromosomes; namely, in the ‘CGTA’ sequence site between the ABC transporter gene and sensory box/GGDEF/EAL protein gene (Fig. 2). By the way, recently, it was experimentally confirmed that *ginABCD* operon, which is located at the left end of the *tox* island, is involved in excision/insertion of the island^13^. However, here, *gin* operon of biovar 6 was found to be truncated (*ginB* is disrupted, and *ginC* and *ginD* are lost), indicating that the *tox* island of biovar 6 might have lost its mobility. It is a future task to clarify whether biovar 6 and biovar 1 independently acquired the *tox* islands or the island was acquired only once at the stage of their common ancestor. Recently, on biovar 1 and biovar 3, elaborate comparative genome analyses have been advanced, which have clarified that horizontal transfers and base substitutions have been actively occurring in these biovars^24,25^. Therefore, while also referring to the results of these analyses, we would like to aim for clarification of the question concerning the *tox* islands.

The size of a gene cluster for coronatine biosynthesis is estimated to be approximately 40 kb^26,27^. Some previous studies have reported that the coronatine gene cluster is located on a megaplasmid in many pathogens (e.g. Psa biovar 2 and *P. syringae* pv. *glycinea* PG4180), but in some cases, it is located on a chromosome directly^14–17,26^. In the present study, it was found that the sequences of the constituent genes of the coronatine gene cluster possessed by biovar 6 were identical or highly homologous to those of their respective homologues possessed by Psa biovar 2 and *P. syringae* pv. *glycinea* PG4180. Moreover, the partition protein gene *parA* was confirmed to exist in the adjacent site to the CFA genes, which are located at the left end of the coronatine gene cluster of biovar 6 (Fig. 3). In addition, the flanking regions, other than *parA*, of the cluster were also found to be homologous to some plasmid-derived sequences (Fig. 3). These results suggest that the coronatine gene cluster in biovar 6 may be located on a plasmid, and that biovar 6 (or its ancestor) may have acquired the ability to produce coronatine through horizontal transfer of the plasmid. However, it could not be determined whether the plasmid carrying the cluster of biovar 6 is the same as that of biovar 2, due to insufficient information.

Although biovar 6 produces two known toxins, it is observed that the disease symptoms caused by biovar 6, appearing on *A. deliciosa* ‘Hayward’ in inoculation tests and infested orchards, are not as severe as those caused by biovar 3^10^, suggesting that the combination and/or balance of various virulence factors may be important for pathogenicity/virulence. For example, some enzymes, phytohormones, and extracellular polysaccharides, in addition to phytotoxins, are also thought to play a role in the invocation of pathogenicity. Moreover, it is known that virulent proteins secreted using secretion apparatus (e.g. type I, II, III, IV, and VI secretion systems) are highly significant in host interaction^28^. Among the various secreted proteins, T3SEs have been the most remarked targets^18,29^. T3SEs are significant in host interaction, which function as virulence factors for susceptible hosts or as avirulence factors for resistant hosts having the corresponding resistant genes^18,29^. Many T3SEs from *Pseudomonas* pathogens have been identified and analysed^18^, and their roles have been revealed considerably. It is supposed that Psa also utilises T3SEs for the establishment of infection to kiwifruit^21^. In this study, 29 T3SE genes were found in biovar 6 (Suppl. Table 2), whose composition was different from that of the other Psa biovars and Pfm (Table 1). Even biovar 3, the closest neighbour to biovar 6 (Fig. 1), possesses a fairly different repertoire of T3SE genes compared to biovar 6. Among T3SE genes—which were previously confirmed to be common to Psa biovars 1, 2, 3, 5 and Pfm^5^—*avrE*, *hopAA1-1*, *hopN1*, *hopS2*, *hopAS1*, *hopR1*, *hopAH1*, and *hopAZ1* are also conserved in biovar 6, whereas *hopI1* is not present in biovar 6. In contrast, *avrRps4* and *hopBI1* are found to be unique to biovar 6. These similarities/differences in T3SE composition might aid our understanding of the pathogenicity and evolution of Psa biovars including biovar 6. For example, Psa/Pfm-conserved T3SE genes (*avrE*, *hopAA1-1*, *hopN1*, *hopS2*, *hopAS1*, *hopR1*, *hopAH1*, and *hopAZ1*) may be associated with an affinity between Psa/Pfm and *Actinidia* spp. In addition, Psa-conserved T3SE genes (*avrD1*, *hopD1*, *hopQ1*, *hopY1*, *hopZ3*, *avrPto5*, and *hopAU1*) may be determinants of the ability to infect kiwifruit systemically and to cause canker and shoot dieback, which are Psa-specific characteristics.

Although the function of the protein encoded by *hopBI1* is not fully known, the function of the AvrRps4 protein derived from *Pseudomonas* pathogens is well researched. For example, AvrRps4 was confirmed to be recognised by the corresponding R protein RPS4 in *Arabidopsis*, which induces RPS4*-*dependent immunity^30^. Furthermore, it was found that on delivery into plant cells via T3SS, AvrRps4 is processed *in planta*, and that the processed AvrRps4 is necessary and sufficient for the activation of effector-triggered immunity (ETI)^31^. Moreover, AvrRps4 was found to target the immunity-associated ED1 protein and induce plant immunity^32,33^. In contrast, AvrRps4 was recently reported to localise to the chloroplast and suppress pathogen/microbe-associated molecular patterns (PAMPs/MAMPs)-triggered immunity (PTI)^34^. In any case, AvrRps4 is thought to be an important effector for plant-microbe interactions. In the case of biovar 6, it is not clear whether this product actually functions in kiwifruit as a virulence/avirulence factor. Since the coronatine synthesis gene cluster and *avrRps4* were found to be present on different plasmids, respectively, it may be possible to change the balance of pathogenicity by introducing these plasmids into other Psa biovars and/or their mutants. Alternatively, by defeating genes of the phytotoxins or T3SEs, the balance of the pathogenicity could be broken, which would aid in clarifying the pathogenic mechanisms of biovar 6.

In this study, by using the genome sequence of biovar 6 as a reference genome for RNA-Seq analysis, we investigated gene expression of biovar 6 during the early stages of host infection. Although the number of reads mapped to the reference genome was small, remarkable change in expression was detected in some pathogenicity-related genes. For example, 23 genes encoding transcriptional regulators, such as a Fis family regulator (BUE60_09075), a TetR family regulator (BUE60_00335), and an XRE family regulator (BUE60_22525), were found to be expressed highly at 4 and/or 8 h after inoculation (Suppl. Table 4), whereas a gene coding for a helix-turn-helix transcriptional regulator (BUE60_20530) was constantly expressed throughout the early stages of host infection (Suppl. Table 4). These transcriptional regulators are thought to respond to changes in the environmental situation, and to induce virulence factors such as phytotoxins and T3SEs^35–37^. In fact, some T3SE genes (e.g. *hopAU1*, *hopQ1* and *hopBI1*) were observed to alter their expression levels, as if they correspond to the expression of genes coding for transcriptional regulators, such as a helix-turn-helix transcriptional regulator (BUE60_20530) and a LysR family regulator (BUE60_21255). Interestingly, approximately 30% of the T3SE genes were induced after 24 h, whereas expression of about 20% T3SE genes was found to vary throughout the periods (Suppl. Table 5, Suppl. Fig. 2). Although 29 T3SE genes were found in biovar 6 (Suppl. Table 2), it is not clear whether these products function in host plants concurrently or separately. However, because these T3SE genes can be categorised into several groups based on their expression patterns (Suppl. Fig. 2), it could be inferred that members belonging to the same group might function cooperatively in hosts during the early stages of infection.

According to the results of RNA-Seq analysis, it is assumed that phaseolotoxin and coronatine might be synthesised at different times: phaseolotoxin synthesis might be suppressed throughout the early stages of host infection (at least up to 24 h later), or its synthesis might be complicatedly regulated; in contrast, about 40% of the coronatine synthesis genes were found to be induced at 4 and/or 8 h after inoculation. Phaseolotoxin has been proved to induce chlorosis around the lesions formed on leaves, by inhibiting the function of ornithine carbamoyltransferase (OCTase), an enzyme involved in arginine biosynthesis^38–40^. Therefore, by using phaseolotoxin, biovar 6 might disrupt the metabolism of hosts and create a favourable environment for itself. Considering the function of phaseolotoxin, there is a possibility that the synthesis of phaseolotoxin from the early stages of infection might not be necessary. On the other hand, coronatine has been reported to have two major functions against plants^27^: one is to suppress the defence response of hosts by disturbing the hormone signals, and the other is to reopen the closed stomata. Because many phytopathogenic bacteria invade plant apoplasts through stomatal pores, host plants are thought to prevent the bacterial invasion by closing the stomatal pores. Then, coronatine-producing bacteria are considered to secure the path of infection by reopening the closed stomata using the function of coronatine^15^. Therefore, it could be inferred that coronatine may be synthesized in the early stages of infection so that biovar 6 can easily invade kiwifruit through its stomatal pores. In order to verify these inferences concerning the temporal and spatial roles of T3SEs and phytotoxins, further studies using Psa biovars with different genetic backgrounds and their properly designed mutants are needed.

Because *avrRps4* is unique to biovar 6 among Psa biovars and Pfm (Table 1), this gene is expected to act as a biovar 6-specific marker. In the present study, we showed that the target region of biovar 6 was amplified by using an *avrRps4*-derived primer set (avrRps4-F1 and avrRps4-R2), and that no amplification was obtained from the other Psa biovars nor Pfm (Fig. 4), indicating that this PCR assay enables us to identify biovar 6 specifically. However, because *avrRps4* is thought to be distributed widely among phyllobacteria, including the *P. syringae* group, and plasmids carrying *avrRps4* might be transferrable, the possibility that this primer set will cause false positives/false negatives is undeniable. Thus, this primer set should be used against bacteria isolated from canker disease symptoms of kiwifruit, in conjunction with Psa-universal primer sets (e.g. ITS- and *hopZ3*-derived primer sets)^41,42^, to avoid incorrect judgement. Through the studies conducted so far, it has been confirmed that the virulence of Psa against kiwifruit is significantly different among biovars^1,4,6,9,10^. Therefore, in order to effectively implement appropriate control measures against canker disease in kiwifruit orchards, clear identification of Psa biovars is required. In addition to other biovar-specific primers and Psa-universal primers^1,5,9,10,43,44^, the biovar 6-specific primer set developed in the present study is essential for complementing the diagnostic procedure and realising accurate and rapid identification. Moreover, by using these biovar-specific primers and Psa-universal primers, we will continue extensive surveillance against the disease, collect pathogens derived from various origins, and conduct their comparative genome analyses in detail, in order to further clarify their diversity and elucidate their centre of origin, evolutionary paths, and pathogenic mechanisms.

## Methods

### Strains and DNA manipulation

The bacterial strains used in this study are listed in Table 2. Culture conditions and procedures for DNA manipulation are the same as those used in our previous study^5^.

**Table 2:** Bacterial strains used in this study.

Strain	Biovar/lineage	Host plant	Location	Isolated year	Reference/Source
***Pseudomonas syringae*** **pv**. ***actinidiae*** **(Psa)**					
MAFF 212130	6	*A. deliciosa*	Nagano, Japan	2015	10
MAFF 212131	6	*A. deliciosa*	Nagano, Japan	2015	10
MAFF 212132	6	*A. deliciosa*	Nagano, Japan	2015	10
MAFF 212133	6	*A. deliciosa*	Nagano, Japan	2015	10
MAFF 212134	6	*A. deliciosa*	Nagano, Japan	2015	10
MAFF 212135	6	*A. deliciosa*	Nagano, Japan	2015	10
MAFF 212136	6	*A. deliciosa*	Nagano, Japan	2015	10
MAFF 212137	6	*A. deliciosa*	Nagano, Japan	2015	10
MAFF 212138	6	*A. deliciosa*	Nagano, Japan	2015	10
MAFF 212139	6	*A. deliciosa*	Nagano, Japan	2015	10
MAFF 212140	6	*A. deliciosa*	Nagano, Japan	2015	10
MAFF 212141	6	*A. deliciosa*	Nagano, Japan	2015	10
MAFF 212142	6	*A. deliciosa*	Nagano, Japan	2015	10
MAFF 211985	1	*A. deliciosa*	Ehime, Japan	2000	4
ICMP 19072	2	*A. chinensis*	Jeonnam, Korea	1997	21
ICMP 18884	3	*A. deliciosa*	Rangiuru, Bay of Plenty, New Zealand	2010	21
ICMP 18744	3	*A. deliciosa*	Rome, Italy	2010	21
ICMP 19079	3	*A. chinensis*	Latina, Italy	2010	21
ICMP 19439	3	*A. deliciosa*	Maule, Chile	2010	21
ICMP 19455	3	*A. deliciosa*	Maule, Chile	2010	21
MAFF 212056	5	*A. chinensis*	Saga, Japan	2012	4
MAFF 212057	5	*A. chinensis*	Saga, Japan	2012	4
***Pseudomonas syringae*** **pv**. ***actinidifoliorum*** (Pfm)*					
CFBP 7812	Lineage 1	*A. chinensis*	Moteuka, New Zealand	2010	6
CFBP 8045	Lineage 1	*A. chinensis*	Western Australia, Australia	1990	6
CFBP 8043	Lineage 2	*A. deliciosa*	Pays de la Loire, France	2011	6
ICMP 18807	Lineage 3	*A. deliciosa*	Tauranga, Bay of Plenty, New Zealand	2010	6
CFBP 7908	Lineage 4	*A. deliciosa*	Aquitaine, France (Nouvelle-Aquitaine, as of 2016)	2011	6
CFBP 8041	Lineage 4	*A. deliciosa*	Pays de la Loire, France	2011	6

*Psa ‘biovar 4’ was transferred to the new pathovar *actinidifoliorum* (Pfm)^6^, which is now divided into four lineages (lineages 1 to 4) (lineages in Pfm are considered to be equivalent to biovars in Psa).

### Genome sequencing and annotation

The strains MAFF 212134 and MAFF 212141 (http://www.gene.affrc.go.jp/databases-micro_search_en.php) were picked up as the representative biovar 6 strains for genome sequencing. Their genomes were sequenced and assembled by using the same protocols used in our previous study^5^, except that some reagents were different as follows; the Ion PGM Hi-Q View OT2 Kit (Thermo Fisher Scientific Inc.), the Ion PGM Hi-Q View Sequencing Kit (Thermo Fisher Scientific Inc.), and a 318 Chip Kit v2 (Thermo Fisher Scientific Inc.) were used in this study instead of the Ion PGM Template OT2 400 Kit, the Ion Sequencing 400 Kit, and a 318 Chip Kit, respectively. The assembled contigs of MAFF 212134 and MAFF 212141 were annotated using the NCBI PGAP (https://www.ncbi.nlm.nih.gov/genome/annotation_prok/), and registered in the nucleotide sequence databases (DDBJ/EMBL/GenBank).

### ANI analysis, comparative genome analysis, and search of pathogenicity-related genes

Average nucleotide identity (ANI) analysis was performed in the same way as our previous study^5^. Reference genome sequences are listed in Suppl. Table 8. When constructing a dendrogram based on ANI values, we used the genomes of *P. syringae* pv. *tomato* (Pst) DC3000 (AE016853) and *P. syringae* pv. *syringae* (Pss) B728a (CP000075) as an outgroup. Comparative genome analyses were performed using Mauve, a multiple genome alignment tool (http://darlinglab.org/mauve/mauve.html)^45^, according to the same protocols as our previous study^5^. In the present study, toxin biosynthesis gene clusters and type III secreted effector (T3SE) genes were selected as representatives of pathogenicity-related genes, and their homologues of biovar 6 were searched and identified by using PGAP, the Mauve tool and tBLASTx analysis in the same way as our previous study^5^.

### Inoculation test

A leaf inoculation test of biovar 6 (MAFF 212134) was conducted according to the method of Cunty *et al*.^6^, with slight modifications. Three 3-month-old *A. deliciosa* ‘Hayward’ specimens were used as hosts. The plants were incubated in a climatic chamber at 20 °C with a relative humidity of more than 90% for 24 h prior to inoculation. A 24-h-old liquid culture of MAFF 212134 was resuspended in sterile water at a concentration of 10^9^ CFU mL^−1^. Leaf inoculation was performed by spraying a suspension of MAFF 212134 on the abaxial side of respective six leaves of three plants. The inoculated plants were placed back in the climatic chamber, with the aforementioned conditions, for 24 h. Three leaves were collected at 0, 4, 8, and 24 h after inoculation for RNA-Seq analysis. After incubation in the climatic chamber, plants were transferred to a closed greenhouse and incubated until symptoms appeared.

### RNA-Seq analysis

Total RNAs were extracted with the RNeasy Plant Mini Kit (Qiagen) from inoculated leaves collected at each time period. By using the RiboMinus Transcriptome Isolation Kit for bacteria (Thermo Fisher Scientific Inc.), rRNAs were almost depleted from the total RNAs. The RNA-Seq library was constructed from sample RNAs with the Ion Total RNA-Seq Kit v2 (Thermo Fisher Scientific Inc.) and Ion Xpress RNA-Seq barcode (Thermo Fisher Scientific Inc.). Subsequently, using the Ion PGM Hi-Q View OT2 Kit on an Ion OneTouch 2 system, RNA-Seq templates were prepared. The templates were sequenced with an Ion PGM Hi-Q View Sequencing Kit and a 318 Chip Kit v2 on an Ion PGM next generation sequencer. Sequence data were assembled and analysed with the CLC Genomics Workbench (Qiagen). The genome sequence of MAFF 212134 was used as the reference for RNA-Seq mapping and assembly of sequence reads. The gene expression value was calculated from the ratio of the number of mapped reads to RPKM at each time, and the subsequent mathematical analysis were all performed by the R program as follows; expression ratio at each time against on 0 h was calculated using the GLM method implemented in the edgeR package according to McCarthy *et al*.^46^. At this time statistical data was obtained by one-way ANOVA. The adjusted p-value and FDR values shown in this study are cited values of these statistical results. And, as is well-known, when the sample parameter is small or the number of iterations is small, the p-value tends to be large^20^. More, because expression comparison is performed for each gene at 0, 4, 8 and 24 h (four variables) in this study, the p-value becomes large. Thus, the second type of error in statistical analysis (Type II error), which produces false negative results, is likely to occur. Therefore, in order to avoid Type II error as much as possible, genes were filtered at 0.1 of permissive FDR value, and the effect size (eta-squared) was calculated and attached on the statistical data of each gene expression. The eta-squared was calculated using the etaSquared method implemented in the lsr package.

### Polymerase chain reaction

Sequences of primers used in this study are as follows: avrRps4*-*F1: 5′-TCAGTTACTCGGCCCCTGCA-3′ and avrRps4*-*R2: 5′-GGCTATTTCGGCTGGATTGC-3′. Colony-direct PCR was performed as described in our previous study^47^. The PCR conditions were as follows: 10 min of pre-denaturation at 95 °C, followed by 40 cycles of 30 s of denaturation at 95 °C, 30 s of annealing at 68 °C, 1 min of extension at 72 °C, and a single final extension of 7 min at 72 °C. The presence and amounts of PCR products were confirmed using agarose gel electrophoresis.

### Accession numbers

The raw sequence data of RNA-seq is available under accession numbers SRR 8569152 - SRR 8569163 and SRR 8569199 - SRR 8569210 in the DDBJ/EMBL/GenBank database. The nucleotide sequence data of MAFF 212134 and MAFF 212141 are available in the DDBJ/EMBL/GenBank database under accession numbers MSBW00000000 and MSBX00000000.

## Supplementary information

Supplementaru Tables and Figures

Supplementary Table 4
