# Supplementary material for: Genome analysis of Pseudomonas syringae pv. actinidiae biovar 6, which produces the phytotoxins, phaseolotoxin and coronatine
Source: Sci Rep. 2019 Mar 7;9:3836. doi: 10.1038/s41598-019-40754-9 (PMC6405952; doi:10.1038/s41598-019-40754-9)
Supplement: Supplementary file 1 — Supplementaru Tables and Figures [file 41598_2019_40754_MOESM1_ESM.pdf]

Genome analysis of *Pseudomonas syringae* pv. *actinidiae* biovar 6, which produces the phytotoxins, phaseolotoxin and coronatine

Takashi Fujikawa and Hiroyuki Sawada

Supplementary Table 1. ANI value between biovar 6 and other biovars

		Biovar 6		Biovar 1											Biovar 2			Biovar 3														Biovar 5		Pfm							Pst
		MAFF21 2134	MAFF21 2141	KW41	PA459	M 302091	ICMP 9617	ICMP 9853	ICMP 9855	ICMP 19068	ICMP 19102	ICMP 19103	ICMP 19104	NCPPB 3871	ICMP 19071	ICMP 19072	ICMP 19073	CFBP 7286	CH 2010-6	ICMP 18708	ICMP 18744	ICMP 18800	ICMP 18801	ICMP 19097	ICMP 19101	ICMP 19439	ICMP 19455	CRAF RU8.43	Shaanxi_ M7	TP1	TP6-1	MAFF 212056	MAFF 212061	ICMP 18804	ICMP 18883	ICMP 19094	ICMP 19095	ICMP 19099	ICMP 19100	ICMP 18807	DC3000
Biovar 6	MAFF212134	100	100	99.62	99.58	99.63	99.62	99.62	99.6	99.6	99.61	99.6	99.6	99.62	99.42	99.43	99.41	99.83	99.84	99.83	99.83	99.83	99.85	99.85	99.84	99.83	99.83	99.82	99.82	99.83	99.83	99.39	99.4	98.21	98.19	98.2	98.2	98.22	98.25	98.22	95.42
	MAFF212141	100	100	99.63	99.58	99.62	99.62	99.62	99.61	99.61	99.62	99.59	99.6	99.62	99.42	99.42	99.42	99.83	99.84	99.83	99.83	99.82	99.84	99.84	99.83	99.83	99.83	99.83	99.82	99.83	99.82	99.39	99.4	98.22	98.17	98.19	98.2	98.19	98.25	98.22	95.41
biovar 1	KW41	99.62	99.63	100	99.99	99.97	100	100	99.99	99.99	99.99	99.98	99.99	100	99.56	99.55	99.56	99.65	99.62	99.62	99.64	99.63	99.64	99.63	99.61	99.65	99.66	99.65	99.62	99.63	99.63	99.55	99.47	98.47	98.44	98.43	98.45	98.45	98.48	98.48	95.98
	PA459	99.58	99.58	99.99	100	99.98	99.99	99.99	99.98	99.97	99.97	99.95	99.98	99.99	99.53	99.56	99.54	99.58	99.57	99.58	99.57	99.56	99.58	99.59	99.58	99.57	99.58	99.57	99.56	99.57	99.56	99.47	99.4	98.46	98.41	98.37	98.46	98.47	98.47	98.48	96
	M302091	99.63	99.62	99.97	99.98	100	99.96	99.97	99.95	99.95	99.97	99.96	99.95	99.97	99.55	99.55	99.57	99.63	99.62	99.62	99.64	99.63	99.62	99.61	99.63	99.66	99.64	99.65	99.6	99.62	99.62	99.54	99.47	98.47	98.43	98.44	98.46	98.48	98.48	98.48	96
	ICMP9617	99.62	99.62	100	99.99	99.96	100	100	99.99	99.98	99.98	99.98	99.98	100	99.55	99.56	99.57	99.64	99.64	99.63	99.65	99.63	99.63	99.63	99.63	99.65	99.65	99.65	99.62	99.63	99.61	99.59	99.46	98.46	98.42	98.42	98.45	98.45	98.47	98.49	96
	ICMP9853	99.62	99.62	100	99.99	99.97	100	100	99.98	99.98	99.98	99.97	99.98	100	99.55	99.54	99.55	99.65	99.63	99.62	99.64	99.63	99.63	99.62	99.63	99.66	99.65	99.65	99.61	99.63	99.62	99.54	99.46	98.46	98.41	98.41	98.44	98.45	98.46	98.46	96
	ICMP9855	99.6	99.61	99.99	99.98	99.95	99.99	99.98	100	99.97	99.97	99.96	99.97	99.98	99.55	99.55	99.55	99.64	99.63	99.61	99.63	99.62	99.63	99.61	99.61	99.65	99.65	99.64	99.61	99.62	99.62	99.52	99.46	98.46	98.4	98.4	98.43	98.46	98.45	98.47	95.99
	ICMP19068	99.6	99.61	99.99	99.97	99.95	99.98	99.98	99.97	100	99.96	99.96	99.97	99.98	99.53	99.55	99.55	99.64	99.61	99.6	99.62	99.6	99.62	99.61	99.6	99.63	99.64	99.63	99.6	99.61	99.63	99.52	99.43	98.48	98.4	98.4	98.44	98.43	98.46	98.46	96
	ICMP19102	99.61	99.62	99.99	99.97	99.97	99.98	99.98	99.97	99.96	100	99.97	99.97	99.98	99.53	99.54	99.54	99.63	99.62	99.61	99.63	99.6	99.62	99.61	99.61	99.63	99.63	99.63	99.59	99.62	99.6	99.52	99.45	98.45	98.41	98.41	98.45	98.46	98.45	98.46	96.01
	ICMP19103	99.6	99.59	99.98	99.95	99.96	99.98	99.97	99.96	99.96	99.97	100	99.96	99.97	99.53	99.53	99.54	99.62	99.61	99.6	99.61	99.59	99.61	99.6	99.61	99.62	99.62	99.63	99.58	99.6	99.59	99.51	99.43	98.44	98.41	98.42	98.45	98.44	98.46	98.46	96
	ICMP19104	99.6	99.6	99.99	99.98	99.95	99.98	99.98	99.97	99.97	99.97	99.96	100	99.98	99.54	99.54	99.55	99.63	99.61	99.61	99.63	99.62	99.62	99.62	99.61	99.63	99.63	99.64	99.61	99.62	99.61	99.52	99.45	98.45	98.4	98.4	98.45	98.45	98.46	98.47	95.97
	NCPPB3871	99.62	99.62	100	99.99	99.97	100	100	99.98	99.98	99.98	99.97	99.98	100	99.56	99.57	99.56	99.65	99.61	99.61	99.64	99.62	99.63	99.63	99.62	99.66	99.66	99.65	99.61	99.62	99.64	99.54	99.46	98.46	98.43	98.41	98.45	98.46	98.48	98.48	95.98
Biovar 2	ICMP19071	99.42	99.42	99.56	99.53	99.55	99.55	99.55	99.55	99.53	99.53	99.53	99.54	99.56	100	99.97	99.88	99.47	99.47	99.46	99.45	99.45	99.48	99.47	99.47	99.45	99.45	99.46	99.46	99.45	99.46	99.49	99.41	98.45	98.4	98.42	98.42	98.43	98.46	98.46	95.96
	ICMP19072	99.43	99.42	99.55	99.56	99.55	99.56	99.54	99.55	99.55	99.54	99.53	99.54	99.57	99.97	100	99.89	99.47	99.48	99.46	99.47	99.48	99.47	99.49	99.49	99.46	99.47	99.47	99.47	99.46	99.47	99.48	99.42	98.45	98.39	98.4	98.42	98.44	98.44	98.45	95.99
	ICMP19073	99.41	99.42	99.56	99.54	99.57	99.57	99.55	99.55	99.55	99.54	99.54	99.55	99.56	99.88	99.89	100	99.47	99.48	99.48	99.46	99.48	99.49	99.48	99.48	99.47	99.47	99.46	99.5	99.47	99.47	99.47	99.36	98.45	98.39	98.39	98.44	98.44	98.46	98.47	96.03
Biovar 3	CFBP7286	99.83	99.83	99.65	99.58	99.63	99.64	99.65	99.64	99.64	99.63	99.62	99.63	99.65	99.47	99.47	99.47	100	99.97	99.97	99.99	99.97	99.96	99.97	99.97	99.98	99.98	99.99	99.96	99.97	99.96	99.4	99.37	98.47	98.39	98.41	98.45	98.46	98.47	98.4	96.04
	CH2010-6	99.84	99.84	99.62	99.57	99.62	99.64	99.63	99.63	99.61	99.62	99.61	99.61	99.61	99.47	99.48	99.48	99.97	100	99.99	99.97	99.99	100	100	100	99.97	99.97	99.96	100	100	99.99	99.44	99.33	98.47	98.43	98.37	98.45	98.45	98.47	98.47	96.02
	ICMP18708	99.83	99.83	99.62	99.58	99.62	99.63	99.62	99.61	99.6	99.61	99.6	99.61	99.61	99.46	99.46	99.48	99.97	99.99	100	99.96	100	100	100	100	99.96	99.97	99.96	100	100	100	99.44	99.33	98.44	98.43	98.39	98.44	98.43	98.48	98.46	96.02
	ICMP18744	99.83	99.83	99.64	99.57	99.64	99.65	99.64	99.63	99.62	99.63	99.61	99.63	99.64	99.45	99.47	99.46	99.99	99.97	99.96	100	99.96	99.97	99.96	99.96	99.98	99.98	99.99	99.96	99.96	99.98	99.45	99.38	98.46	98.38	98.42	98.44	98.46	98.47	98.46	96.02
	ICMP18800	99.83	99.82	99.63	99.56	99.63	99.63	99.63	99.62	99.6	99.6	99.59	99.62	99.62	99.45	99.48	99.48	99.97	99.99	100	99.96	100	100	100	100	99.96	99.97	99.96	100	100	100	99.43	99.34	98.46	98.43	98.39	98.45	98.45	98.47	98.46	96.02
	ICMP18801	99.85	99.84	99.64	99.58	99.62	99.63	99.63	99.63	99.62	99.62	99.61	99.62	99.63	99.48	99.47	99.49	99.96	100	100	99.97	100	100	99.99	99.99	99.97	99.96	99.97	100	100	100	99.43	99.35	98.47	98.44	98.39	98.47	98.47	98.48	98.47	96.06
	ICMP19097	99.85	99.84	99.63	99.59	99.61	99.63	99.62	99.61	99.61	99.61	99.6	99.62	99.63	99.47	99.49	99.48	99.97	100	100	99.96	100	99.99	100	99.99	99.96	99.97	99.96	100	100	100	99.46	99.35	98.47	98.45	98.39	98.44	98.46	98.47	98.48	96.02
	ICMP19101	99.84	99.83	99.61	99.58	99.63	99.63	99.63	99.61	99.6	99.61	99.61	99.61	99.62	99.47	99.49	99.48	99.97	100	100	99.96	100	99.99	99.99	100	99.96	99.96	99.96	100	100	100	99.44	99.33	98.5	98.46	98.39	98.48	98.49	98.49	98.49	96.09
	ICMP19439	99.83	99.83	99.65	99.57	99.66	99.65	99.66	99.65	99.63	99.63	99.62	99.63	99.66	99.45	99.46	99.47	99.98	99.97	99.96	99.98	99.96	99.97	99.96	99.96	100	100	99.98	99.96	99.96	99.97	99.47	99.39	98.45	98.38	98.41	98.43	98.44	98.47	98.44	96
	ICMP19455	99.83	99.83	99.66	99.58	99.64	99.65	99.65	99.65	99.64	99.63	99.62	99.63	99.66	99.45	99.47	99.47	99.98	99.97	99.97	99.98	99.97	99.96	99.97	99.96	100	100	99.98	99.97	99.97	99.96	99.47	99.37	98.45	98.38	98.41	98.44	98.45	98.47	98.45	96.02
	CRAFRU8.43	99.82	99.83	99.65	99.57	99.65	99.65	99.65	99.64	99.63	99.63	99.63	99.64	99.65	99.46	99.47	99.46	99.99	99.96	99.96	99.99	99.96	99.97	99.96	99.96	99.98	99.98	100	99.95	99.96	99.97	99.46	99.38	98.47	98.4	98.41	98.45	98.47	98.47	98.48	96.03
	Shaanxi_M7	99.82	99.82	99.62	99.56	99.6	99.62	99.61	99.61	99.6	99.59	99.58	99.61	99.61	99.46	99.47	99.5	99.96	100	100	99.96	100	100	100	100	99.96	99.97	99.95	100	100	100	99.43	99.33	98.47	98.43	98.37	98.44	98.44	98.48	98.46	96.08
	TP1	99.83	99.83	99.63	99.57	99.62	99.63	99.63	99.62	99.61	99.62	99.6	99.62	99.62	99.45	99.46	99.47	99.97	100	100	99.96	100	100	100	100	99.96	99.97	99.96	100	100	100	99.44	99.33	98.48	98.43	98.39	98.43	98.45	98.47	98.46	96.03
	TP6-1	99.83	99.82	99.63	99.56	99.62	99.61	99.62	99.62	99.63	99.6	99.59	99.61	99.64	99.46	99.47	99.47	99.96	99.99	100	99.98	100	100	100	100	99.97	99.96	99.97	100	100	100	99.44	99.36	98.45	98.44	98.39	98.45	98.45	98.47	98.45	96.01
Biovar 5	MAFF212056	99.39	99.39	99.55	99.47	99.54	99.59	99.54	99.52	99.52	99.52	99.51	99.52	99.54	99.49	99.48	99.47	99.4	99.44	99.44	99.45	99.43	99.43	99.46	99.44	99.47	99.47	99.46	99.43	99.44	99.44	100	100	98.43	98.33	98.39	98.43	98.42	98.45	98.43	95.94
	MAFF212061	99.4	99.4	99.47	99.4	99.47	99.46	99.46	99.46	99.43	99.45	99.43	99.45	99.46	99.41	99.42	99.36	99.37	99.33	99.33	99.38	99.34	99.35	99.35	99.33	99.39	99.37	99.38	99.33	99.33	99.36	100	100	98.15	98.05	98.11	98.18	98.17	98.19	98.19	95.33
Pfm	ICMP18804	98.21	98.22	98.47	98.46	98.47	98.46	98.46	98.46	98.48	98.45	98.44	98.45	98.46	98.45	98.45	98.45	98.47	98.47	98.44	98.46	98.46	98.47	98.47	98.5	98.45	98.45	98.47	98.47	98.48	98.45	98.43	98.15	100	99.97	99.97	99.97	99.97	99.99	99.13	96.03
	ICMP18883	98.19	98.17	98.44	98.41	98.43	98.42	98.41	98.4	98.4	98.41	98.41	98.4	98.43	98.4	98.39	98.39	98.39	98.43	98.43	98.38	98.43	98.44	98.45	98.46	98.38	98.38	98.4	98.43	98.43	98.44	98.33	98.05	99.97	100	99.86	99.95	99.96	99.97	99.11	96.04
	ICMP19094	98.2	98.19	98.43	98.37	98.44	98.42	98.41	98.4	98.4	98.41	98.42	98.4	98.41	98.42	98.4	98.39	98.41	98.37	98.39	98.42	98.39	98.39	98.39	98.39	98.41	98.41	98.41	98.37	98.39	98.39	98.39	98.11	99.97	99.86	100	99.96	99.96	99.97	99.1	96.02
	ICMP19095	98.2	98.2	98.45	98.46	98.46	98.45	98.44	98.43	98.44	98.45	98.45	98.45	98.45	98.42	98.42	98.44	98.45	98.45	98.44	98.44	98.45	98.47	98.44	98.48	98.43	98.44	98.45	98.44	98.43	98.45	98.43	98.18	99.97	99.95	99.96	100	99.96	99.97	99.1	96.05
	ICMP19099	98.22	98.19	98.45	98.47	98.48	98.45	98.45	98.46	98.43	98.46	98.44	98.45	98.46	98.43	98.44	98.44	98.46	98.45	98.43	98.46	98.45	98.47	98.46	98.49	98.44	98.45	98.47	98.44	98.45	98.45	98.42	98.17	99.97	99.96	99.96	99.96	100	99.98	99.13	96.07
	ICMP19100	98.25	98.25	98.48	98.47	98.48	98.47	98.46	98.45	98.46	98.45	98.46	98.46	98.48	98.46	98.44	98.46	98.47	98.47	98.48	98.47	98.47	98.48	98.47	98.49	98.47	98.47	98.47	98.48	98.47	98.47	98.45	98.19	99.99	99.97	99.97	99.97	99.98	100	99.13	96.06
	ICMP18807	98.22	98.22	98.48	98.48	98.48	98.49	98.46	98.47	98.46	98.46	98.46	98.47	98.48	98.46	98.45	98.47	98.4	98.47	98.46	98.46	98.46	98.47	98.48	98.49	98.44	98.45	98.48	98.46	98.46	98.45	98.43	98.19	99.13	99.11	99.1	99.1	99.13	99.13	100	96.02
Pst	DC3000	95.42	95.41	95.98	96	96	96	96	95.99	96	96.01	96	95.97	95.98	95.96	95.99	96.03	96.04	96.02	96.02	96.02	96.02	96.06	96.02	96.09	96	96.02	96.03	96.08	96.03	96.01	95.94	95.33	96.03	96.04	96.02	96.05	96.07	96.06	96.02	100

Supplementary Table 2. Reference table of T3SE genes in gene locus tag of biovar 6

	MAFF 212134	MAFF 212141
avrRpm1	BUE60_10450	BUE61_22645
avrE1	BUE60_16630	BUE61_10755
hopM1	BUE60_16620	BUE61_10765
hopAA1-1	BUE60_23665	BUE61_13935
hopN1	BUE60_23680	BUE61_13920
hopS2	BUE60_19875	BUE61_27870
hopBB1-2	BUE60_22740	BUE61_28575
hopAW1	BUE60_22760	BUE61_28555
hopAY1	BUE60_03125	BUE61_20570
avrB4	BUE60_28445	BUE61_28825
avrD1	BUE60_25205	BUE61_28050
hopD1	BUE60_25165	BUE61_14980
hopQ1	BUE60_25170	BUE61_14975
hopA1	BUE60_08965	BUE61_14055
hopY1	BUE60_06595	BUE61_26390
hopZ3	BUE60_19375	BUE61_18645
hopAS1	BUE60_13800	BUE61_13270
hopAE1	BUE60_29005	BUE61_28845
hopW1	BUE60_10130	BUE61_22900
hopR1	BUE60_10120	BUE61_22910
hopAH1	BUE60_21995	BUE61_02640
hopAI1	BUE60_21990	BUE61_02635
avrPto5	BUE60_21190	BUE61_23395
hopAZ1	BUE60_01140	BUE61_28180
hopAV1	BUE60_19190	BUE61_20995
hopAU1	BUE60_24650	BUE61_29020
hopE1	BUE60_28965	BUE61_27535
avrRps4	BUE60_15715	BUE61_17920
hopBI1	BUE60_29210	BUE61_29115

Supplementary Table 3. List of samples showing the number of total reads and mapped reads obtained in RNA-Seq

Samples	Number of total reads	Number of mapped reads	Average length (bp)
0h-1	458,793	183,800	175.4
0h-2	238,460	86,004	195.7
0h-3	244,786	91,996	190.3
0h-4	577,020	149,438	187.2
0h-5	405,960	95,005	204.8
0h-6	389,811	88,366	199.6
4h-1	135,964	15,781	206.4
4h-2	155,017	15,781	209.1
4h-3	318,130	80,574	194.8
4h-4	270,513	14,508	217.3
4h-5	337,378	36,022	223.3
4h-6	536,935	70,418	209.1
8h-1	313,066	23,956	171.2
8h-2	256,477	17,582	166.2
8h-3	239,532	45,301	174.3
8h-4	695,856	21,081	187.8
8h-5	455,887	15,486	179.3
8h-6	440,221	38,604	192.3
24h-1	274,106	52,851	166.1
24h-2	114,994	12,304	206.6
24h-3	197,315	30,687	208.0
24h-4	430,066	47,935	176.9
24h-5	241,802	12,958	217.9
24h-6	438,887	31,324	220.0

Total reads were filtered with a Phred score cut-off of <20 for quality preservation.
Mapped reads were specifically corresponded to MAFF 212134 genome.

Genome analysis of *Pseudomonas syringae* pv. *actinidiae* biovar 6, which produces the phytotoxins, phaseolotoxin and coronatine
Takashi Fujikawa and Hiroyuki Sawada

Supplementary Table 4. List of expressed genes of biovar 6 during the early stages of host infection.

See the other file “Suppl Table 4.xslx”

Supplementary Table 5. List of expressed T3SE genes of biovar 6 during the early stages of host infection.

Locus tag	Gene	LogFC 4h	LogFC 8h	LogFC 24h	LogCPM	p-value	FDR	η^2
BUE60_19190	*hopAV1*	-0.543273858	-1.766260847	-2.853028208	7.582618505	4.00E-10	1.15E-08	0.747732913
BUE60_15715	*avrRps4*	-0.50466047	-1.781430377	-3.238599402	7.084053938	1.25E-06	2.03E-05	0.587424099
BUE60_16620	*hopM1*	-1.460005874	-1.371063155	-0.304498945	10.39229195	0.001081096	0.007754994	0.660100272
BUE60_19040	*hopAH1*	-1.351851012	-1.473924844	-0.004454316	6.520864132	0.031472498	0.120485204	0.618634834
BUE60_22760	*hopAW1*	-0.992247065	-4.402955919	1.705412125	5.261584658	0.066647125	0.208465588	0.16221863
BUE60_25170	*hopQ1*	-0.613046939	0.353208882	1.209269302	5.661119129	0.078779294	0.23584958	0.212959214
BUE60_21995	*hopAH1*	0.238251738	-1.51943246	-3.998355313	4.644909957	0.093015019	0.266361192	0.275625005
BUE60_29005	*hopAE1*	-0.419936149	-0.158746443	1.21768865	5.181912305	0.187500561	0.430909299	0.179655764
BUE60_13800	*hopAS1*	-0.257255908	-1.519873452	0.792461949	4.996811612	0.214951239	0.473710031	0.2864189
BUE60_25205	*avrD1*	-0.40535304	-1.606254249	-1.595885745	6.187905953	0.293340996	0.572379301	0.517881347
BUE60_25165	*hopD1*	-0.226283672	-0.345520167	-1.274693155	5.61682373	0.329381463	0.612281898	0.55935817
BUE60_19375	*hopZ3*	0.958115674	-2.881995545	0.946618123	4.549403995	0.36260019	0.651264549	0.110584723
BUE60_21990	*hopAI1*	-1.44368242	-3.949854514	-0.483087037	4.508236197	0.408267157	0.700747805	0.315056972
BUE60_06595	*hopY1*	-1.753739864	-4.173778124	0.016231595	4.641224524	0.449367097	0.739345315	0.180282051
BUE60_16630	*avrE1*	-0.30687391	-0.620071902	0.402156916	5.839721829	0.44987299	0.739554414	0.647105447
BUE60_10130	*hopW1*	-0.972764432	-1.539890196	-0.969923663	5.836767164	0.483457896	0.770448711	0.525620771
BUE60_08965	*hopA1*	-0.810249014	-1.212236501	-0.465681465	6.398248604	0.507320385	0.793852309	0.533641879
BUE60_10120	*hopR1*	-0.567983009	-0.565999417	0.075098343	5.541419434	0.523424738	0.805568185	0.56417777

BUE60_10755	*hopAV1* (incomplete)	-0.466250203	-0.769321352	-0.48651316	6.392853746	0.58350025	0.855111871	0.580954279
BUE60_23665	*hopAA1*	0.906028047	-0.364096299	0.578014882	5.535644555	0.584568944	0.855164462	0.172187768
BUE60_19875	*hopS2*	1.782351918	-2.325980753	1.496245046	4.561641987	0.598614625	0.867521553	0.172436862
BUE60_03125	*hopAY1*	-1.882638565	-1.637500116	0.09806001	5.061242324	0.603292641	0.871679638	0.224092643
BUE60_10450	*avrRpm1*	-0.792014902	-1.218590727	-0.398070612	5.535482775	0.731717959	0.960814973	0.462156396
BUE60_29210	*hopBI1*	-0.486874441	1.414402276	1.355029188	4.768981308	0.76653941	0.982499429	0.045838257
BUE60_24650	*hopAU1*	0.265111968	0.776644694	0.384025841	5.848829287	0.794161	0.997606666	0.081670956
BUE60_19045	*hopAH1* (incomplete)	-0.452565513	-0.018286622	-1.384112404	4.955884622	0.863498852	1	0.132284793
BUE60_22740	*hopBB1*	-0.298275825	-1.018048425	0.263458326	5.366961581	0.860412964	1	0.152811399
BUE60_23680	*hopN1*	-0.973932166	-0.897556088	-0.298817793	5.262007386	0.865784708	1	0.449629896
BUE60_28965	*hopE1*	0.595734385	-0.521179462	0.3737961	5.459845	0.871192924	1	0.177587348

LogFC means the log2 fold-change against 0 h sample.

LogCPM means the average log2 counts per million over all samples.

η^2 value of each gene means effective size.

A gene with pink background was continuously expressed during the early stages of infection.

Genes with blue background were continuously suppressed during the early stages of infection.

A gene with orange background was induced at 4 h and/or 8 h after inoculation.

Genes with yellow background were induced only at 24 h after inoculation.

Genes with green background were induced at various periods.

avrRps4 and *hopBI1* are biovar 6 specific effector genes.

Supplementary Table 6. List of expressed phaseolotoxin (*argK-tox* gene cluster) genes of biovar 6 during the early stages of host infection.

Locus tag	Gene product	LogFC 4h	LogFC 8h	LogFC 24h	LogCPM	p-value	FDR	η^2
BUE60_02345	hypothetical protein	-0.830653527	-0.43907058	-3.893210782	6.271461017	0.000894543	0.006625642	0.536455767
BUE60_02350	hypothetical protein	-1.894332129	-6.496222141	-2.392796016	5.580458042	0.00742228	0.039287246	0.592004981
BUE60_02355	aspartate aminotransferase family protein, *argD*	-0.393250358	-1.49995974	-1.630753916	5.660961542	0.239098024	0.507623498	0.648573309
BUE60_02360	cyclase	-1.118534113	-0.403568703	-0.000633436	10.53991852	0.269832009	0.545969408	0.55923923
BUE60_02365	dehydrogenase	-0.26381605	-1.710012518	0.364883082	5.438270211	0.573986841	0.847815425	0.118339311
BUE60_02370	fatty acid desaturase, *desI*	-2.208378002	-4.476365449	-0.656801477	4.652985635	0.183328963	0.425711006	0.315567952
BUE60_02375	deoxycytidine triphosphate deaminase	-2.714342797	1.966446563	-2.714342797	4.410863524	0.649124851	0.904188328	0.095026808
BUE60_02380	dCTP deaminase	0.948461565	0.568526211	1.459254183	4.955270515	0.947709542	1	0.006570378
BUE60_02385	pyruvate, phosphate dikinase	-1.866945789	-1.257738162	0.153931873	5.072882383	0.096249152	0.272392621	0.412302013
BUE60_02390	hypothetical protein	-0.350353403	0.56196664	0.111259352	5.619970406	0.942282394	1	0.101037754
BUE60_02395	hypothetical protein	-1.770639443	0.200733939	-4.895010953	4.99286454	0.103762327	0.285975487	0.30236705
BUE60_02400	hypothetical protein	-2.149370533	-1.073367398	-0.808996088	4.857934924	0.518119071	0.802731392	0.477365236
BUE60_02405	HAD family hydrolase	-3.658800777	-0.554660953	-3.658800777	4.358684612	0.423978138	0.718768872	0.164584759

BUE60_02410	amidinotransferase, *amtA*	0.294790561	0.207853687	-1.625826818	5.362712624	0.456663924	0.74474926	0.241686572
BUE60_02415	hypothetical protein	-1.652547149	-4.542602325	-0.890650876	4.677620933	0.134574298	0.343760525	0.505163525
BUE60_02420	adenylyl-sulfate kinase	-4.54619866	1.129402271	-0.805388042	5.113842775	0.042117533	0.149213461	0.217562722
BUE60_02425	hypothetical protein	-1.127110163	-3.928220697	-0.292251834	4.541581786	0.218475989	0.477896269	0.414332052
BUE60_02430	hypothetical protein	-1.554230575	-3.792718349	-1.119085135	4.395864728	0.408285945	0.700747805	0.328552248
BUE60_02435	hypothetical protein	-3.540948109	-0.027476868	-3.540948109	4.368584164	0.423141217	0.71795272	0.168206775
BUE60_23870	ornithine carbamoyltransferase; OCTase, *argK*	-1.78114744	-1.671741507	-1.035941803	10.14703344	0.002519475	0.015889967	0.741157882
BUE60_23875	hypothetical protein	-1.006524047	-1.00849209	-3.574281345	6.752767021	2.51E-06	3.77E-05	0.820467584
BUE60_23880	hypothetical protein	-1.229254175	-1.479460789	-2.819926888	6.185281576	0.041816088	0.148417663	0.6364218
BUE60_23885	hypothetical protein	0.100965436	0.037911775	-0.329265634	6.074991488	0.962807015	1	0.201766937

logFC means the log2 fold-change against 0 h sample.

LogCPM means the average log2 counts per million over all samples.

η^2 value of each gene means effective size.

A gene with pink background was continuously expressed during the early stages of infection.

Genes with blue background were continuously suppressed during the early stages of infection.

Genes with orange background were induced at 4 h and/or 8 h after inoculation.

Genes with yellow background were induced only at 24 h after inoculation.

A gene with green background was induced at various periods.

Supplementary Table 7. List of expressed coronatine genes of biovar 6 during the early stages of host infection.

Locus tag	Gene product	Group	LogFC 4h	LogFC 8h	LogFC 24h	LogCPM	p-value	FDR	η^2
BUE60_10635	crotonyl-CoA carboxylase/reductase *cfa9*	cfa	-0.195163418	-0.491202281	-2.534025083	7.477374	3.26E-05	0.00037	0.474415
BUE60_10640	hypothetical protein *cfa8*	cfa	-1.42216523	-3.547134392	-3.096752497	6.184432	0.044812	0.156465	0.535402
BUE60_10645	polyketide synthase *cfa7*	cfa	0.008904115	-0.091924101	-1.517805283	6.665468	0.006206	0.033745	0.525568
BUE60_10650	polyketide synthase *cfa6*	cfa	-0.084185939	-0.281050162	-1.226973381	7.093432	0.006377	0.034412	0.689801
BUE60_10655	coronafacic acid synthetase *cfa5*	cfa	-0.376895699	-0.993850815	-0.82732505	7.266606	0.148164	0.369994	0.527445
BUE60_10660	coronafacic acid synthetase *cfa4*	cfa	-1.033570453	-1.742442386	-4.083666976	9.097996	4.16E-13	1.84E-11	0.698555
BUE60_10665	beta-ketoacyl synthase *cfa3*	cfa	-0.514582852	-0.802861621	-2.444947032	8.081012	6.21E-10	1.73E-08	0.786446
BUE60_10670	beta-hydroxyacyl-ACP dehydratase *cfa2*	cfa	0.312195847	-0.814265453	-8.666649252	8.186743	2.78E-21	3.93E-19	0.534148
BUE60_10675	coronafacic acid synthetase *cfa1*	cfa	0.222843006	0.122413905	-6.862800283	6.736594	0.000593	0.004641	0.255937
BUE60_10680	coronafacic acid synthetase *cfl*	cfa	0.108875371	-0.38083922	-4.844439114	9.080239	5.81E-35	3.37E-32	0.646415
BUE60_22770	DNA-binding response regulator *corR*	cma	-0.149585371	-0.999156408	-2.02981715	8.219264	1.11E-05	0.000146	0.632245
BUE60_22775	histidine kinase *corS*	cma	-0.557970554	-1.412294115	-2.422715678	7.560922	0.0002	0.001803	0.699229
BUE60_22780	response regulator *corP*	cma	0.621638596	1.47152524	0.94372696	5.665229	0.904697	1	0.020076
BUE60_22785	hypothetical protein	cma	0.147255096	-1.25007986	-2.545903273	6.834695	0.007612	0.040111	0.693565
BUE60_22790	IS66 family transposase	cma	-0.95094595	-1.326294769	-2.479804366	7.583151	1.55E-05	0.000195	0.755662

BUE60_22795	transposase	cma	-2.831429252	3.246962439	3.130748778	5.535011	0.052944	0.176155	0.17275
BUE60_22800	hypothetical protein	cma	-5.361562849	-5.361562849	1.282279367	5.621543	0.002435	0.015405	0.211274
BUE60_22805	cma gene	cma	-5.415606905	-5.415606905	-5.415606905	4.721865	0.040375	0.144694	0.422448
BUE60_22810	cma gene	cma	0.216186598	0.619507193	-7.609120963	7.563442	6.40E-09	1.51E-07	0.46083
BUE60_22815	coronamic acid synthetase *cmaE*	cma	0.155191506	-0.553802609	-3.329246637	9.193487	3.29E-17	2.42E-15	0.700784
BUE60_22820	coronamic acid synthetase *cmaA*	cma	0.034952843	-0.652066897	-3.497172013	9.267226	6.53E-18	5.65E-16	0.714698
BUE60_22825	*cmaB*	cma	0.005089106	-0.478675411	-5.911732989	10.25566	3.79E-42	4.39E-39	0.901928
BUE60_22830	coronamic acid synthetase *cmaC*	cma	0.555579129	-0.355980794	-2.347546552	7.816935	0.008812	0.04515	0.522017
BUE60_22835	coronamic acid synthetase thioesterase *cmaT*	cma	-0.184333808	-0.441779053	-5.197966871	8.149573	9.48E-17	6.39E-15	0.711703
BUE60_28700	coronamic acid synthetase *cmaU*	cma	-0.574617651	-1.00824071	-2.950269941	7.902832	9.31E-07	1.54E-05	0.786878

logFC means the log2 fold-change against 0 h sample.

LogCPM means the average log2 counts per million over all samples.

η^2 value of each gene means effective size.

A gene with pink background was continuously expressed during the early stages of infection.

Genes with blue background were continuously suppressed during the early stages of infection.

Genes with orange background were induced at 4 h and/or 8 h after inoculation.

A gene with yellow background was induced only at 24 h after inoculation.

A gene with green background was induced at various periods.

Supplementary Table 8. Bacterial reference genomes used in ANI analysis

Strain	Biovar/ Lineage	GenBank accession number
Pseudomonas syringae pv. *actinidiae* (Psa)		
ICMP 9853	1	ANJB00000000
ICMP 9855	1	AOKB00000000
ICMP 19068	1	AOJX00000000
ICMP 19102	1	AOKA00000000
ICMP 19103	1	AOJQ00000000
ICMP 19104	1	AOJZ00000000
Kw41	1	AGNP00000000
PA459	1	AGNQ00000000
MAFF 302091	1	AEAL00000000
ICMP 9617	1	AFTH00000000
NCPPB 3871	1	AFTF00000000
ICMP 19071	2	AOJS00000000
ICMP 19072	2	AOJW00000000
ICMP 19073	2	AOJR00000000
CFBP 7286	3	AGNO00000000
CH2010-6	3	AGUH00000000
ICMP 18708	3	ANJC00000000
ICMP 18744	3	ANGD00000000
ICMP 18800	3	ANJD00000000
ICMP 18801	3	AOKQ00000000
ICMP 19097	3	AOKN00000000
ICMP 19101	3	AOKM00000000
ICMP 19439	3	ANJM00000000
ICMP 19455	3	ANJK00000000
CRAFRU8.43	3	AFTG00000000
Shaanxi_M7	3	ANJJ00000000
TP1	3	ANJG00000000
TP6-1	3	ANJH00000000
MAFF 212056	5	BBWG00000000
MAFF 212061	5	NKQU00000000

Pseudomonas syringae pv. *actinidifoliorum* (Pfm)		
ICMP 18804	1	ANJE00000000
ICMP 18883	1	AOKH00000000
ICMP 19094	1	AOKJ00000000
ICMP 19095	1	AOKI00000000
ICMP 19098	1	AOKE00000000
ICMP 19099	1	AOKD00000000
ICMP 19100	1	AOKC00000000
ICMP 18807	3	ANJL00000000
Pseudomonas syringae pv. *syringae* (Pss)		
B728a		CP00075
Pseudomonas syringae pv. *tomato* (Pst)		
DC3000		AE016853

Supplementary Figure 1

a

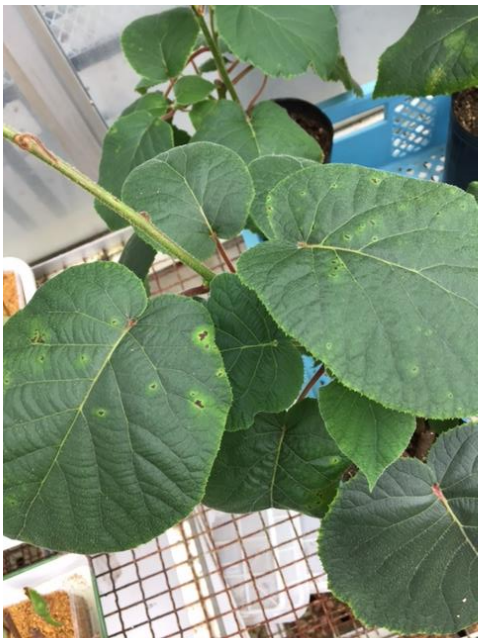

b

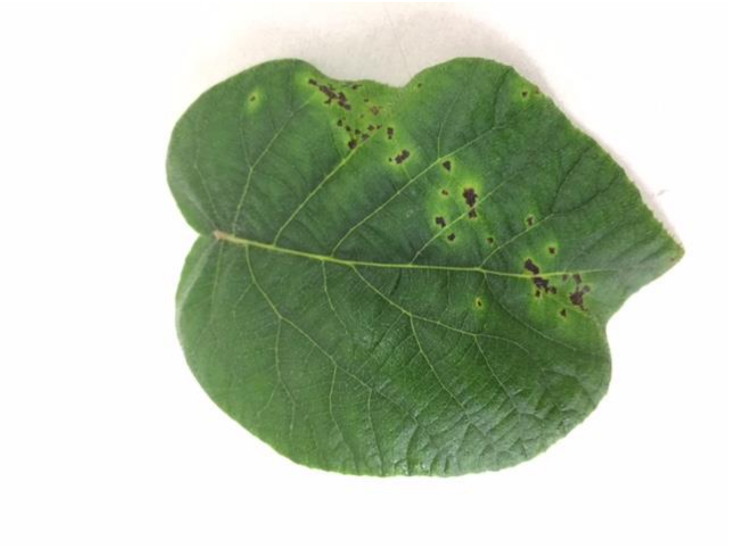

Disease symptom by spray inoculation of biovar 6.

Bacterial suspension of biovar 6 (MAFF 212134) was spray inoculated to *A. deliciosa* ‘Hayward’. Typical lesions of leaves were observed to be formed gradually from one week after inoculation. Leaves of (a) and (b) are lesion developed at 2 weeks after inoculation.

Supplementary Figure 2

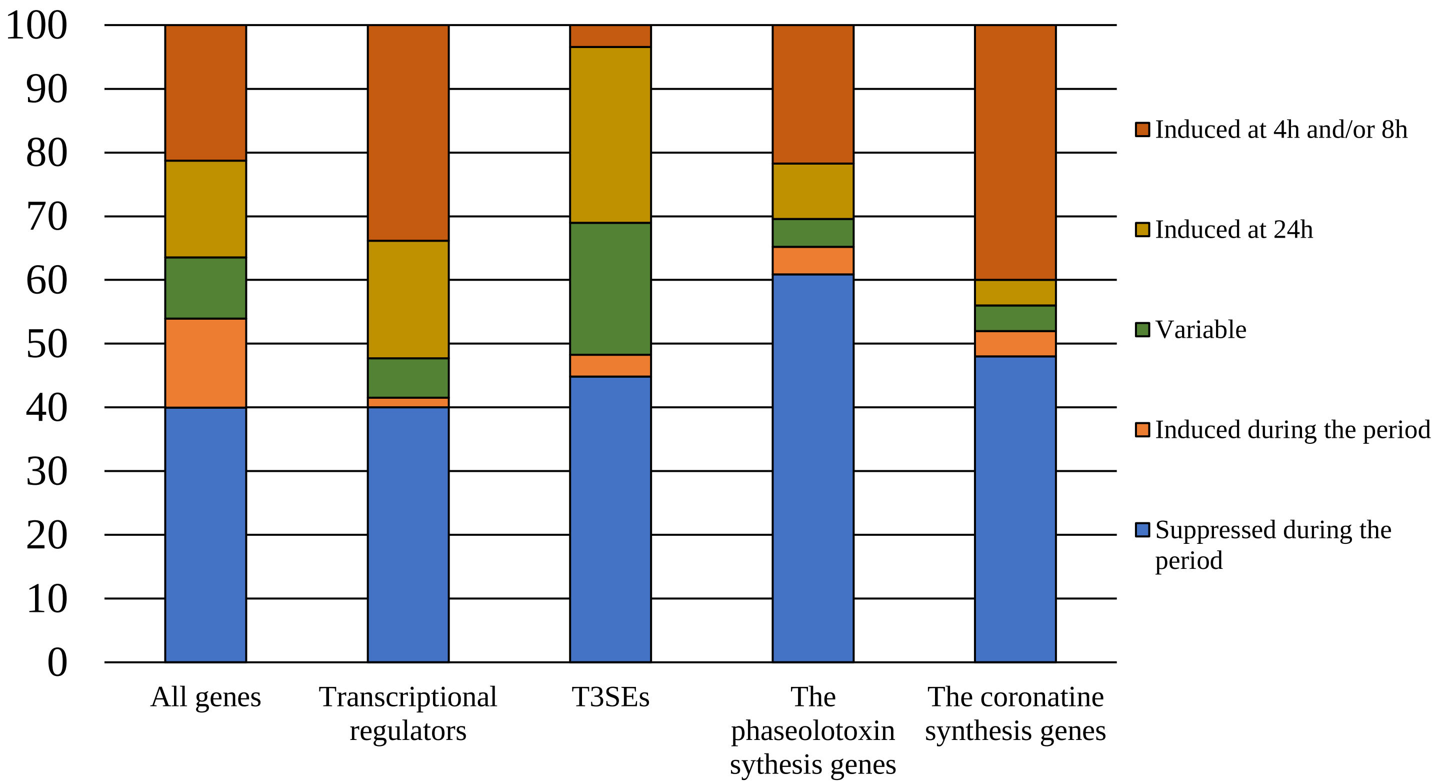

Proportion of expression pattern for each gene group

The expressed genes of biovar 6 during the early stages of host infection obtained by RNA-seq analysis were divided according to the types of gene (all genes, transcriptional regulators, T3SEs, the phaseolotoxin synthesis genes, and the coronatine synthesis genes), and indicated the proportion of expressed pattern. The value of vertical axis means percent.
